# Prediction of Monoclonal Antibody Pharmacokinetics in Pediatric Populations Using PBPK Modeling and Simulation

**DOI:** 10.3390/pharmaceutics17070884

**Published:** 2025-07-05

**Authors:** Chiara Zunino, Virginie Gualano, Haiying Zhou, Viera Lukacova, Maxime Le Merdy

**Affiliations:** 1 Phinc Development, 36 Rue Victor Basch, 91300 Massy, France; 2 Simulations Plus, Inc., P.O. Box 12317, Research Triangle Park, NC 27709, USA

**Keywords:** PBPK, monoclonal antibody, translation, pediatrics

## Abstract

**Background**: Accurately determining pediatric dosing is essential prior to initiating clinical trials or administering medications in routine clinical settings. In children, ethical considerations demand careful evaluation of both safety and effectiveness. Typically, dosing recommendations for therapeutic proteins, such as monoclonal antibodies (mAbs), are derived from adult dosages using body weight as a scaling factor. However, this method overlooks key physiological and biochemical distinctions between pediatric and adult patients. Therefore, this could lead to the underexposure of mAbs, limiting their efficacy in this population. Additional methods are necessary to predict pediatric doses mechanistically. For small molecules, physiologically based pharmacokinetic (PBPK) models have been extensively used to predict pediatric doses based on physiological age-related changes and enzymes/transporters ontogeny. This study aims to evaluate the ability of PBPK models to predict mAbs’ pediatric exposure. **Methods**: Three mAbs were used for model development and validation: bevacizumab, infliximab, and atezolizumab. The PBPK models were built using GastroPlus^©^ Biologics module. For each mAb, the PBPK model was developed based on observed data in healthy and/or patient adults. Then, the physiological parameters were scaled to describe the pediatric physiology to predict exposure to the pediatric populations. Predicted plasma concentration–time courses were overlaid with reported observed data to assess the ability of the PBPK model to predict pediatric exposure. **Results**: Results showed that PBPK models accurately predicted pediatric pharmacokinetics for mAbs. **Conclusions**: This research marks a significant step in validating mechanistic extrapolation methods for biologics exposure prediction in children using PBPK models.

## 1. Introduction

Nowadays, monoclonal antibodies (mAbs) constitute the majority of biological drugs used in clinical practices [1]. These large molecules (molecular weight~150 kDa) play a key role in treating pathologies such as autoimmune diseases, neurological disorders, or cancers. Those diseases affect multiple populations, including pediatric patients. However, developing mAbs for pediatric use requires careful and prospective consideration of differences in both disease etiology and age-mediated modification of the pharmacokinetics (PK) and pharmacodynamics (PD) of mAbs. A primary challenge is selecting accurate doses for pediatric patients. Pediatric mAb doses are often extrapolated from adult doses using allometric scaling based on body weight or surface area [2]. However, this approach does not consider the rapidly changing physiological and biochemical modifications between children and adults affecting PK and PD of mAbs. This could potentially lead to inadequate exposure of mAbs, limiting their efficacy in the pediatric population. To address these limitations, alternative approaches that mechanistically predict pediatric doses are essential. Modeling and simulations offer valuable tools to support clinicians in optimizing mAb dose selection for pediatric patients.

Physiologically based pharmacokinetic (PBPK) models have long supported the development and regulatory assessment of small molecules [3]. However, the underlying mechanisms affecting the PK of small molecules and mAbs are significantly different. Hence, new PBPK models have been developed to specifically support the development of mAbs [4]. Their structure has evolved to integrate the key physiological phenomenon affecting mAbs absorption, distribution, and elimination through catabolism. Among those, the tissue distribution of mAbs mediated either by pinocytosis or convection flows across endothelial pores can be described [5]. Furthermore, the affinity to the Neonatal Fc receptor (FcRn) plays an essential role in protecting endogenous and exogenous antibodies against catabolism reactions degrading the mAbs in the endosomal space of the endothelial cells [4,6]. Including the mAbs-FcRn complex in the PBPK models algorithm allowed the mechanistic description of the typical observed long half-life of mAbs [1]. Finally, implementation of target-mediated drug disposition (TMDD) allowed the PBPK models to describe the nonlinear clearance of mAbs at low doses [7,8].

PBPK models have already demonstrated their abilities to predict pediatric doses of small molecules based on physiological age-related changes, enzymes/transporters ontogeny, as well as the composition of tissues and organ growth, among other changes [9]. Using a similar strategy as what is typically done for small molecules (developing the model in adults and then predicting PK in pediatrics), PBPK models have been developed and showed accurate predictions of some PK of mAbs for the pediatric population using multiple modeling platforms [10,11,12,13], demonstrating the great promises of this approach. In a recent review, Lim et al. pointed out how pediatric PBPK studies for mAbs were still limited despite the increasing appreciation of their utility in accounting for how the ontogeny of pediatric physiology could affect the PK of mAbs [14]. Indeed, by the time of their review in May 2023, there were only seven pediatric PBPK models published, and these pediatric PBPK studies were limited to 10 mAbs relative to the 39 mAbs used for pediatric populations in 2023 [14]. Hence, validating pediatrics PBPK models either using additional case studies or using different modeling platforms is a necessity to support the development and regulatory assessment of mAbs indicated for pediatric patients.

In this context, this study aims to demonstrate the ability of the PBPK model implemented in GastroPlus^©^ to predict the PK of mAbs in pediatric populations. Bevacizumab (BEV), infliximab (INF), and atezolizumab (ATE) were used as validation case studies.

This study includes: (1) the development and validation of PBPK models in healthy adult subjects and adult patients following single or multiple intravenous (IV) administrations of BEV, INF, or ATE; and (2) the validation of the ability of the PBPK model to predict pediatrics exposure for those APIs.

## 2. Methods

### 2.1. Case Studies

BEV, INF, and ATE were selected based on data availability in both adult and pediatric populations. These mAbs are all administered intravenously. At therapeutic doses, these exposures of mAbs are dose proportional.

BEV is a recombinant humanized IgG1 mAb targeting the circulating VEGF-A protein. This mAb is used to reduce the microvascular growth of tumor blood vessels, and thus limits the blood supply of tumors. It is indicated for metastatic colorectal cancer, first-line non-squamous non-small cell lung cancer, recurrent glioblastoma, and metastatic renal cell carcinoma. The usual dosing schedule is 5 mg/kg every two weeks for adults [15].

INF is a humanized IgG1 mAb directed against soluble and membrane-bound tumor necrosis factor-alpha (TNF-α). It is used to treat autoimmune conditions (including Crohn’s disease and rheumatoid arthritis). The approved weight-based induction and maintenance dose is the same for pediatric and adult patients: 5 mg/kg for inflammatory bowel disease [16].

ATE is a humanized IgG1 mAb targeting soluble and membrane-bound PD-L1 (human programmed death-ligand 1) expressed on immune cells and tumor cells. It blocks PD-1 (program death protein 1) mediated inhibitory signals. This monoclonal antibody is approved for hepatocellular carcinoma, urothelial carcinoma, melanoma, and small cell/non-small cell lung cancer. The usual therapeutic dose is 15 mg/kg with a maximum of 1200 mg administered every three weeks [17].

### 2.2. Software and Model Structure

GastroPlus (version 9.9 Simulation Plus Inc., Lancaster, CA, USA) biologics module was used for simulation of BEV, INF, and ATE biodistribution in healthy adults, adult patients, and pediatric patients. The PBPK model structure integrates key elements affecting the PK of mAbs. Each organ is divided into three compartments: vascular, endosomal, and interstitial volumes. The model is parameterized using both drugs’ specific and physiological parameters. Drug-specific parameters are vascular reflection coefficient, lymph reflection coefficient, FcRn binding, and antigen binding. For the physiological parameters, tissues’ specific antigen concentration, size, composition, blood perfusion rate, lymph flow rate, FcRn concentration, and antigen concentration are implemented in the PBPK model. The PBPK model also accounts for the presence of circulating endogenous antibodies. A complete description of the model structure and the underlying equations are presented in Supplementary Material S1.

### 2.3. Pediatric Translation

**Figure 1:**
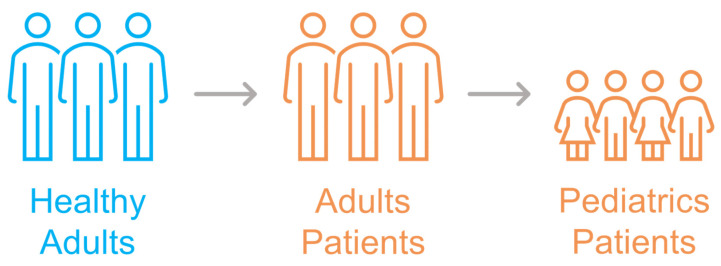
PBPK-based translation method to predict PK of mAbs in pediatric patients.

Figure 1 presents the PBPK-based translation method used to predict PK of mAbs in pediatric patients. To simulate the PK from individual clinical studies, study population demographics (sex, mean age, mean body weight, mean body mass index [BMI], disease state) of each study were matched as closely as possible, dependent upon available information about the age, body weights, BMIs, and number of males and females in each clinical study. PBPK models were initially developed and validated using PK data in healthy adults. The validated PBPK models were then calibrated to account for diseases’ impact on PK of mAbs. Once the PBPK models were validated for adult patients, the physiological parameters were scaled to match the pediatric physiology to predict exposure in the pediatric population. Clinical studies used for models’ development and validation are presented in Supplementary Material S2. For ATE, no PK data in healthy adults were publicly available. Hence the model was directly validated using PK data in adult patients. In all cases, the model was deemed acceptable if the simulated plasma concentration time courses could be overlaid with observed data, and if no systematic mispredictions could be identified. When applicable, comparisons between observed and simulated PK metrics were made to evaluate PBPK model performance.

### 2.4. PBPK Parameters

Initial simulations were performed using GastroPlus default parameters. To enhance the model predictions in adult populations (healthy adults for BEV and INF, or adult patients for ATE), three parameters have been optimized for each mAb: the vascular and lymphatic reflection coefficients and the degradation rate constant of unbound endosomal exogenous mAbs (kdeg). TMDD has been integrated into the PBPK model solely for BEV. TMDD parameters were extracted from the literature. The soluble antigen VEGF-A has been integrated in the vascular compartment of the BEV PBPK model with input parameters from fixed based on literature values [18,19,20]. For both INF and ATE, the TMDD was not considered as the observed PK data demonstrated linear PK across the therapeutic range in both adult and pediatric populations.

BEV PBPK model was adjusted to account for disease conditions in adult and pediatric patients. Circulating VEGF-A concentration in patients is known to be two- to ten-fold higher compared to healthy subjects [18,19,20]. Therefore, in the PBPK model, VEGF-A expression has been multiplied by two-fold for both adult and pediatric patient populations.

Clinical data suggests INF clearance is increased in patients affected by inflammatory diseases [21]. Hence, for the INF PBPK model, kdeg has been increased by 1.5-fold for both adult and pediatric patient populations.

Once the models were validated for adult patients, predictions for pediatric populations could be performed. The specific parameters of the drugs fitted using adult PK data (Table 1) were kept constant for the pediatric PBPK models. Only the physiological parameters were scaled to match pediatric physiology to predict exposure in a pediatric population.

## 3. Results

### 3.1. Bevacizumab

For BEV, a total of nine clinical studies were identified in the literature: six in healthy adults (Bev.Ad.H.1 to Bev.Ad.H.6), two in cancer patients (Bev.Ad.P.1 and Bev.Ad.P.2), and one in pediatric cancer patients (Bev.Ped.P) (information about the clinical studies are provided in Supplementary Material S2). For this case study, the PBPK-based translation method used to predict PK of mAbs in pediatric patients is presented in Figure 1.

**Figure 2:**
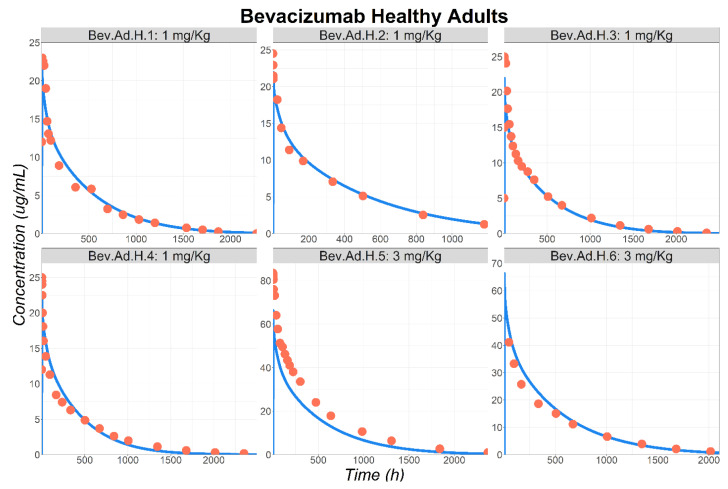
Observed (circles) and simulated (lines) BEV plasma concentration–time course in healthy subjects following IV administration. Model development was done based on Bev.Ad.H.1: 1 mg/kg to 27.6 YO subjects [22]. Model validation was achieved using the following studies: Bev.Ad.H.2: 1 mg/kg to 27 YO subjects [23]; Bev.Ad.H.3: 1 mg/kg to 31 YO subjects [24]; Bev.Ad.H.4: 1 mg/kg to 23 YO subjects [25]; Bev.Ad.H.5: 3 mg/kg to 39.5 YO subjects [26]; Bev.Ad.H.6: 3 mg/kg to 39.8 YO subjects [27]. Abbreviations: YO: years old.

First, the model was validated to describe BEV PK in healthy subjects. One study was used for model development (Bev.Ad.H.1 [22]) and all the others were used for external validation (Bev.Ad.H.2 to Bev.Ad.H.6) [23,24,25,26,27]. To describe the BEV Cp–time profile following its IV administration, the vascular and lymphatic reflection coefficients were fitted to 0.99 and 0.63 (unitless). Furthermore, the endosomal clearance parameter (kdeg) was fitted to 1.24 × 10^4^ 1/day. For this mAb, the effect of TMDD on its clearance is noticeable based on the observed plunging characteristic shape of the elimination phase [28]. Therefore, the TMDD was included in the PBPK model. To parameterize the TMDD, circulating VEGF-A’s expression was fixed to 1.96 × 10^−6^ μmol/mL-plasma in healthy subjects based on literature data [18,19,20]. All the other TMDD parameters were fixed based on literature information (Table 1). Once BEV PBPK model parameters were calibrated, BEV Cp–time profiles following its IV administration to healthy volunteers were well described (Figure 2). Fold differences between observed and simulated PK parameters are provided in Supplementary Material S3.

**Figure 3:**
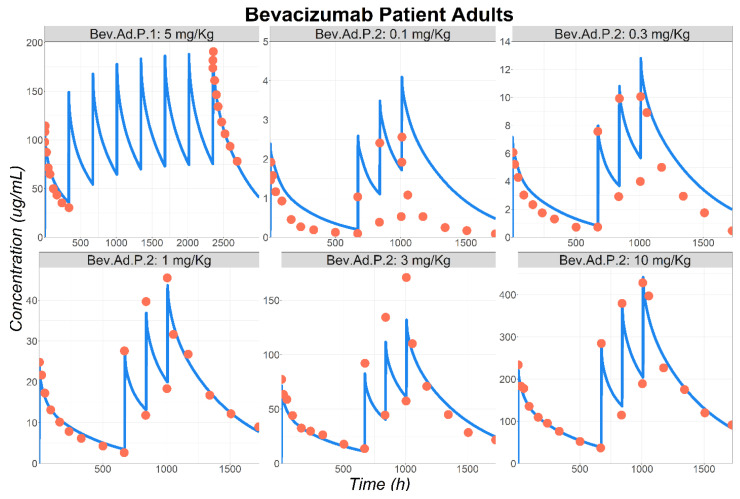
Observed (circles) and simulated (lines) BEV plasma concentration–time course in patients following IV administration. Bev.Ad.P.1: 10 mg/kg to 56 YO patients [29], Bev.Ad.P.2: 0.1 to 10 mg/kg to 51 YO patients [30]. Abbreviations: YO: years old.

Once the model was validated in healthy volunteers, it was adapted to adult cancer patients by increasing VEGF-A circulating concentration by two-fold based on literature information [18,19,20]. Following this model adjustment, the observed Cp–time profiles following BEV IV administration to cancer patients (Bev.Ad.P.1 and Bev.Ad.P.2 [29,30]) were well-predicted by the model (Figure 3). However, it should be noted that the observed data following the administration of 0.1 mg/kg [30] were slightly over-predicted by the model. Based on those overall positive results, the PBPK model was deemed acceptable and used to predict BEV Cp–time following its IV administration to the pediatric population.

**Figure 4:**
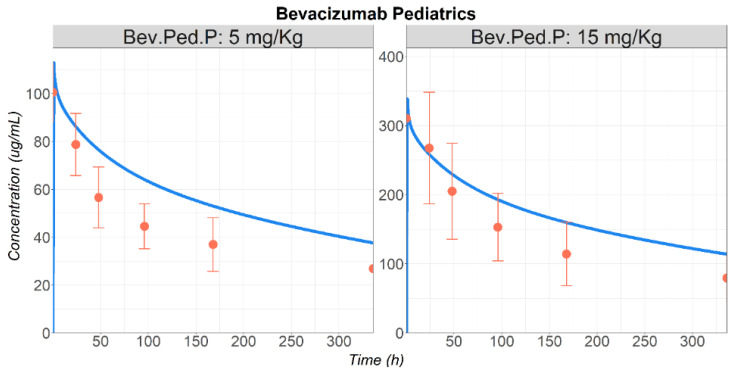
Observed (circles) and simulated (lines) BEV plasma concentration–time course in pediatric patients following IV administration of 5 or 15 mg/kg. Simulations were performed for a virtual 13 YO, 53.65 Kg, male patient (Study Bev.Ped.P [10]). Abbreviations: YO: years old.

For pediatric predictions, Basu et al. (Bev.Ped.P [10]), reported mean PK profiles for pediatric patients receiving either 5 or 15 mg/kg. Hence, the predictions were performed for a representative individual by creating a physiology for a virtual 13 years old, 53.65 Kg, male patient, based on the published demographic information [31,32]. All the other drug-specific parameters, as well as circulating VEGF-A concentrations, were assumed similar to the ones used to describe BEV PK in adult patients. The PBPK model provided reasonable predictions of BEV Cp–time profiles in the pediatric population at both doses (Figure 4).

### 3.2. Infliximab

For INF, a total of twelve clinical studies were identified in the literature: two in healthy adults (Inf.Ad.H.1 and Inf.Ad.H.2), eight in inflammatory disease patients, and two in pediatric patients (Inf.Ped.P.1 and Inf.Ped.P.2) (information about the clinical studies are provided in Supplementary Material S2). For this case study, the PBPK-based translation method used to predict PK of mAbs in pediatric patients is also presented in Figure 1.

One of the studies performed on adult healthy subjects was used for model development (Inf.Ad.H.1 [33]) and the other one was used for external validation (Inf.Ad.H.2 [34]). To describe the INF Cp–time profile following its IV administration, the vascular and lymphatic reflection coefficients were fitted to 0.99 and 0.60 (unitless). Additionally, kdeg was fitted to 1.34 × 10^4^ 1/day. Overall, the model could accurately predict the observed data following IV administration of 5 mg/kg (simulation results, and fold differences between observed and simulated PK parameters are presented in Supplementary Material S3).

Once the model was validated in healthy volunteers, it was adapted to adult patients affected by inflammatory diseases by increasing the endosomal clearance (kdeg) by 1.5-fold to account for the inflammation effect on mAb clearance [21]. Following this model adjustment observed Cp–time profiles following INF IV administration to patients (studies Inf.Ad.P.1 to Inf.Ad.P.8 [35,36,37,38,39,40]) were overall well-predicted by the model (simulation results are presented in Supplementary Material S3). The INF PBPK model was deemed acceptable and used to predict INF Cp–time following its IV administration to the pediatric population.

**Figure 5:**
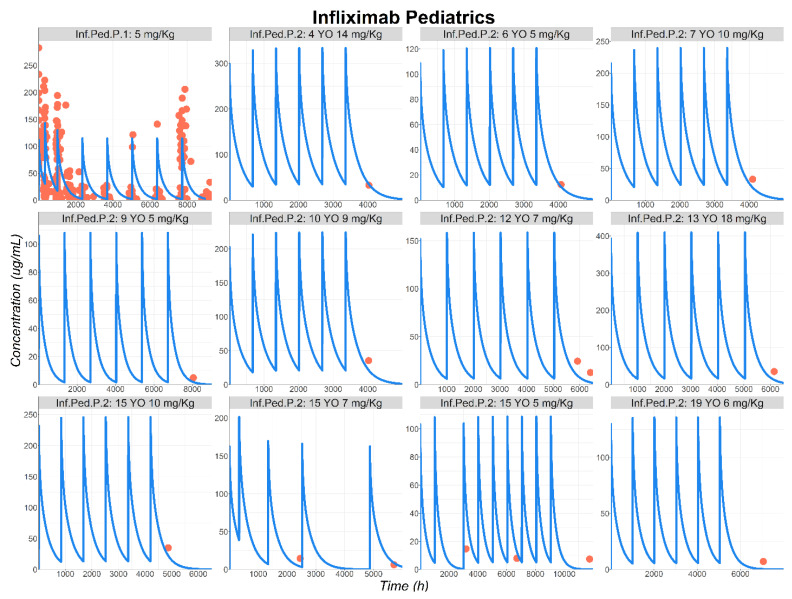
Observed (circles) and simulated (lines) INF plasma concentration–time course in pediatric patients following IV administration of: 5 mg/kg (Study Inf.Ped.P.1:13 YO, 42 Kg, male patient [40]); pediatric patients ranging from 4 to 19 YO receiving multiple doses from 5 to 18 mg/kg (Study Inf.Ped.P.2 [11]). Abbreviations: YO: years old.

For pediatric predictions, Fasanmade et al. (Inf.Ped.P.1 [40]) reported individual INF Cp–time profiles for a population ranging from 6 to 18 years old. However, the observed data were lumped together, and it was not possible to sort them based on age or body weight. Hence, to describe the observed data, a virtual representative pediatric patient was generated using the published median demographic information for age and body weight [41]. The administered dose was specified to be 5 mg/kg resulting in a 210 mg dose for the virtual pediatric patient. The model well-described the observed data, confirming the ability of the PBPK model to predict PK of mAbs in pediatric populations following its validation using adult PK data (Figure 5).

Chang et al. (Inf.Ped.P.2 [11]) presented individual INF Cp–time profiles for pediatric patients ranging from 4 to 19 years old. Individual dose and dosing schedules were set based on published information. To perform the simulations, a virtual representative individual was used for each age group by creating specific physiologies, based on published demographic information [42,43]. All the drug-specific parameters were kept constant between adults and pediatric patients. Final simulations for INF concentration time courses in pediatric patients are presented in Figure 5. Although only a single data point was available for certain age groups, limiting the extent of PBPK model validation, the overall data description and the various simulated profiles were deemed acceptable. Therefore, for the two pediatric studies, the PBPK model provided reasonable predictions of INF Cp–time profiles in the pediatric population (Figure 5).

### 3.3. Atezolizumab

For ATE, two clinical studies in adult and pediatric cancer patients were identified in the literature (Ate.Ad.P and Ate.Ped.P) (information about the clinical studies are provided in Supplementary Material S2). Therefore, for this case study, the PBPK-based translation method used to predict PK of mAbs in the pediatric population was directly performed using the model developed in adult cancer patients.

The first study used describes ATE PK in adult cancer patients following its IV administration at five different strengths (1, 3, 10, 15, and 20 mg/kg: Ate.Ad.P [44]). The 15 mg/kg strength (typical clinical strength) was used for model development and the other strengths were used for external validation. The vascular and lymphatic reflection coefficients were fitted to 0.99 and 0.29 (unitless) to describe the ATE Cp–time profile following its IV administration. Furthermore, kdeg was fitted to 1.8 × 10^4^ 1/day in adult cancer patients. Once the PBPK model was adjusted, ATE Cp–time profiles following its IV administration to adult cancer patients were well described for all strengths [44] (simulation results and fold differences between observed and simulated PK parameters are presented in Supplementary Material S3). Based on these results, the PBPK model was fully validated and used to predict ATE Cp–time following its IV administration to the pediatric population.

**Figure 6:**
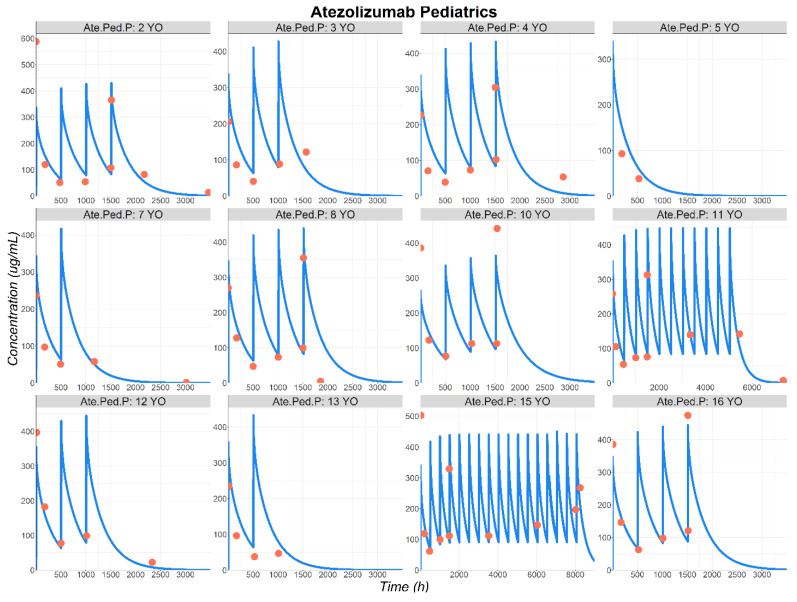
Observed (circles) and simulated (lines) ATE plasma concentration–time course in pediatric patients following IV administration (15 mg/kg or 1200 mg: Ate.Ped.P [13]). Abbreviations: YO: years old.

Huang et al. (Ate.Ped.P [13]) presented the mean ATE Cp–time profile for pediatric patients ranging from 2 to 16 years old. The administered dose was 15 mg/kg with a maximum of 1200 mg per patient. To perform the simulations, a virtual representative pediatric patient was used for each age group by creating specific physiologies, based on published demographic information [45,46]. All the drug-specific parameters were kept constant between adults and pediatric patients. Final simulations for ATE concentration time courses in pediatric patients are presented in Figure 6. Despite having a sparse dataset for certain age groups, overall, the data description was correct. Therefore, the PBPK model provided reasonable predictions of ATE Cp–time profiles in the pediatric population (Figure 6).

## 4. Discussion

Pediatric diseases, ranging from common infections to chronic conditions and cancers, require specialized medical care tailored to the unique physiological characteristics of children. Biologics play an increasing role in the treatment of pediatric patients, offering targeted therapies for conditions that were previously difficult to manage using solely small molecules. Among those biologics, mAbs have become essential in the treatment of autoimmune disorders and certain types of cancer. Between 2018 and 2023, the number of mAbs with pediatric indication has risen from eleven to thirty-nine. PBPK models have already proven effective in predicting pediatric doses of small molecules by accounting for age-related physiological changes and their impact on drugs’ ADME. The current study builds upon the work done in the field of small molecule prediction for pediatric patients to demonstrate the ability of the PBPK model implemented in GastroPlus to predict the PK of mAbs in pediatric populations.

Following intravenous administration, mAbs undergo phases of distribution and elimination through catabolism. Among the physiological differences between adults and pediatric patients, tissue size and composition significantly influence the distribution of mAbs across plasma and extracellular volumes. These variances are incorporated into the PBPK models used for pediatric simulations. In most studies, the observed pediatric pharmacokinetic profiles were accurately predicted, validating the embedded algorithm’s ability to predict mAbs distribution in pediatric populations. However, in some instances, observed C_max_ were over- or under-predicted (e.g., prediction for ATE in two- and ten-year-old subgroups). All pediatric prediction data utilized in this research were derived from patients affected by either cancer or inflammatory autoimmune diseases. Both conditions are associated with edema: solid tumors may obstruct lymphatic circulation, resulting in fluid buildup, while inflammation increases blood vessel permeability, leading to fluid leakage and tissue swelling. Additionally, certain treatments for these conditions (e.g., corticosteroids, and chemotherapy) are known to induce edema. Consequently, the body composition of pediatric patients with these diseases may differ from the known one of healthy children. This may significantly impact mAbs distribution and contribute to some of the over- or under-predictions of C_max_ in this population.

As outlined in the introduction, the FcRn system plays a key role in the catabolic elimination rate of mAbs. In the current iteration of the PBPK software, the relative expression of FcRn based on age is not considered. Nevertheless, Barber et al. [47] elucidated a declining trend in FcRn abundance from neonatal to adult levels. Furthermore, Hardiansyah et al. [48] evaluated the effects of the FcRn developmental pharmacology on the pharmacokinetics of mAbs in pediatric subjects using a minimal PBPK model. This study estimated a decrease in FcRn expression between one and twenty years old. However, Han et al. [32] conducted a population PK analysis for bevacizumab and demonstrated the absence of clearance differences between pediatric and adult patients. Using a comparable methodology, the estimated clearances for INF and ATE were similar for children and adults [40,46]. These findings are corroborated by the analysis presented in this research project. Indeed, the elimination rate is overall well-predicted across pediatric patients using the PBPK model, even though it does not consider potential age-related differences in FcRn expression level. One possible explanation is that although FcRn expression levels decrease between neonates and adults, it remains above a saturation threshold and does not directly impact mAb elimination rates. An additional analysis was conducted using a global parameter sensitivity analysis (PSA) on all parameters related to the FcRn system, including Kon and Koff at pH 6, as well as individual FcRn concentrations in each tissue. The PSA results indicate that FcRn density across tissues has minimal or no impact on mAbs PK metrics. In contrast, the FcRn binding parameters at pH 6 (Kon and Koff) do influence PK outcomes, as shown in Supplementary Material S4. However, this simulated impact does not compromise the conclusions of the study, since these parameters are correlated with the fitted endosomal clearance. Therefore, if different binding affinity values were used for FcRn, the endosomal clearance parameter would be adjusted accordingly to obtain comparable simulation results.

Another key factor known to affect the elimination rate of mAbs is the TMDD. The concept of TMDD was highlighted in 1994 by Levy [49] to describe a phenomenon in which a drug binds with high affinity and to a significant extent (relative to the administered dose) to its pharmacologic target site (receptor or enzyme). This interaction can be reflected in PK of mAbs at low circulating concentrations, when the target is not yet saturated, causing a non-linear PK [28]. In the present study, TMDD was considered only for BEV, which is known to have a nonlinear clearance for the dose range used in this analysis that was adequately reproduced by the PBPK model. For both INF and ATE, the TMDD was not integrated into their respective PBPK models, as the clinical doses used were sufficiently high for this phenomenon not to occur. The target expression level is one of the key parameters needed to incorporate the TMDD into a PBPK model. For the BEV case study, VEGF-A expression level in healthy and cancer adults was parametrized based on literature data. However, this information could not be identified for cancer pediatric patients. It was assumed that the VEGF-A expression level was constant for all cancer patients, either pediatric or adult patients. This hypothesis may not be true. However, the clinical doses used in pediatric patients [10] were above the target saturation threshold and the TMDD should have a limited impact on BEV overall clearance. To test this hypothesis, a PSA on VEGF-A circulating concentration in pediatric patients was done. This PSA demonstrates that modifying the circulating VEGF-A concentration at clinical doses has no impact on BEV exposure (Supplementary Material S4). Hence, the BEV PBPK model was deemed acceptable for this research project and no further improvements were needed to support the study conclusions.

Clinical trials conducted on pediatric patients for INF and ATE described their systemic exposure at steady-state. The PK of these mAbs is characterized by a relatively long half-life, allowing for less frequent dosing compared to small molecule drugs. Hence, these clinical trial lengths extend for multiple months. When using a PBPK model to predict the PK of a small molecule over multiple months in pediatric subjects, it is common practice to adjust the physiological parameters to account for the physiological changes occurring during this period. This is particularly true for young children for whom physiology evolves rapidly. However, for INF and ATE case studies, the PBPK models used only one physiology to describe a representative virtual pediatric patient across the length of the trial. As mentioned previously, these patients are affected by diseases impacting their physiology. Furthermore, some comedications may have been administered to them, also affecting their physiology. Due to the variability in individual treatment responses, potential side effects, and other unknown factors, adjusting the PBPK physiology to account for patients’ evolution during the clinical trials was not feasible. This could explain some variations between observed and predicted concentrations at steady-state.

The common clinical practice for adjusting mAbs doses in pediatric patients typically involves extrapolating adult doses based on body weight or body surface area. This approach, known as allometric scaling, assumes the existence of a universal body weight-based scaling relationship. However, it does not address physiological and biochemical differences between children and adults, such as organ maturity, enzyme activity, or immune system development [2]. In this context, PBPK models represent an advanced methodology as it integrates the known physiological changes happening between childhood and adulthood. Furthermore, as these models describe all of the key physiological factors affecting PK of mAbs (e.g., blood and lymph flows, FcRn-mediated salvage, target affinity, and nonlinear pharmacokinetic [1,9]), they are ideally positioned to describe disease effects and make a step towards individualized medicine. This approach is not feasible using common allometric scaling solely based on body weight. Therefore, PBPK models will be increasingly used to predict pediatric dosing and improve safety and efficacy for this special population.

In conclusion, this research project has seen the development and validation of PBPK models for three mAbs administered intravenously based on clinical data in healthy subjects and adult patients. These models were successfully applied to predict mAbs clinical exposure in pediatric patients. This proposed methodology to extrapolate pediatric systemic PK based on a PBPK model validated using clinical data in adults can support the drug development paradigm as well as the current clinical practice, by predicting the safe and efficacious dose in this pediatric population.

## Figures and Tables

**Table 1:** Parameter values implemented in PBPK models for bevacizumab, infliximab, and atezolizumab.

Parameter	Bevacizumab (Healthy/Patient)		Infliximab (Healthy/Patient)		Atezolizumab (Patient)	
Molecular weight (kDa)	149	[15]	149.1	[16]	145	[17]
Vascular Reflection Coefficient	0.99	Fitted	0.99	Fitted	0.99	Fitted
Lymphatic Reflection Coefficient	0.63	Fitted	0.60	Fitted	0.29	Fitted
Kon FcRn pH 6 (1/μM/day)	8000	GP default	8000	GP default	8000	GP default
Koff FcRn pH 6 (1/day)	500	GP default	500	GP default	500	GP default
Endosomal clearance (1/day)	1.24 × 10^4^	Fitted	1.34 × 10^4^/ 2.00 × 10^4^	Fitted	1.8 × 10^4^	Fitted
TMDD integrated	Yes		No		No	
Kon VEGF-A (1/μM/day)	30	[20]	NA			
Koff VEGF-A (1/day)	0.006	[20]				
Kint (1/day)	1					
Expression of VEGF-A (μmol/mL-plasma)	1.96 × 10^−6^/ 3.86 × 10^−6^	[18,19,20]				
Kdeg complex (1/day)	0.173					
Ksyn VEGF-A (μmol/g-plasma/day)	0.34 × 10^−6^/ 0.67 × 10^−6^	[18,19,20]				

GP = GastroPlus. NA: not applicable.

## Data Availability

The original contributions presented in this study are included in the article/supplementary material. Further inquiries can be directed to the corresponding author.

## Supplementary Materials

The following supporting information can be downloaded at: https://www.mdpi.com/article/10.3390/pharmaceutics17070884/s1, Materials S1. GastroPlus^®^ Biologic Module—Figure S1: Schematic representation of physiologically based pharmacokinetic model of disposition of biologic drugs; Figure S2: Schematic representation of individual tissue compartments. Materials S2—Table S1: List of studies used for development and qualification of PBPK models in current study. Materials S3—Figure S3: Observed (circle) and simulated (lines) infliximab plasma concentration–time course in healthy subjects following IV administration (5 mg/kg); Figure S4: Observed (circle) and simulated (lines) infliximab plasma concentration–time course in patients following IV administration; Figure S5: Observed [44] (circles) and simulated (lines) atezolizumab plasma concentration–time course in patients following IV administration; Table S2: C_max_ and AUC_0–t_, observed, simulated, and prediction fold error for studies used in baseline PBPK model validation. Materials S4—Table S3: Parameter sensitivity analysis settings to assess effect of FcRn on mAbs PK. PSA was done using Study Inf.Ped.P.1; Table S4: Parameter sensitivity analysis results for C_max_ (Study Inf.Ped.P.1); Table S5: Parameter sensitivity analysis results for AUC (Study Inf.Ped.P.1); Figure S6: Simulated BEV plasma concentration–time course in pediatric patients following IV administration of 5 mg/kg (Study Bev.Ped.P) with varying concentration of circulating VEGF-A. References [10,11,13,22,23,24,25,26,27,29,30,31,33,34,35,36,37,38,39,40,42,43,44,46,50,51,52,53] are cited in the supplementary materials.
