# Supplementary material for: Prediction of Monoclonal Antibody Pharmacokinetics in Pediatric Populations Using PBPK Modeling and Simulation
Source: Pharmaceutics. 2025 Jul 5;17(7):884. doi: 10.3390/pharmaceutics17070884 (PMC12298552; doi:10.3390/pharmaceutics17070884)
Supplement: Supplementary file 1 [file pharmaceutics-17-00884-s001.zip › pharmaceutics-3700365-supplementary.pdf]

Prediction of Monoclonal Antibody Pharmacokinetics in Pediatric Populations Using PBPK Modeling and Simulation

Chiara Zunino [1], Virginie Gualano [1], Haiying Zhou [2], Viera Lukacova [2] and Maxime Le Merdy [2,*]

1: Phinc Development, 36 rue Victor Basch, 91300 Massy, France

2: Simulations Plus, Inc., P.O. Box 12317, Research Triangle Park, NC 27709, USA

*Corresponding author: Maxime Le Merdy

Email: maxime.lemerdy@simulations-plus.com

Supplementary Material S1

GastroPlus® Biologic Module

The biologic module includes a physiologically based pharmacokinetic (PBPK) model for simulation of biological drug absorption, distribution, and clearance, including monoclonal antibodies (mAb) administered as intravenous bolus or intravenous infusion. A schematic diagram of how the different organs are connected to one another is shown in Figure S1.

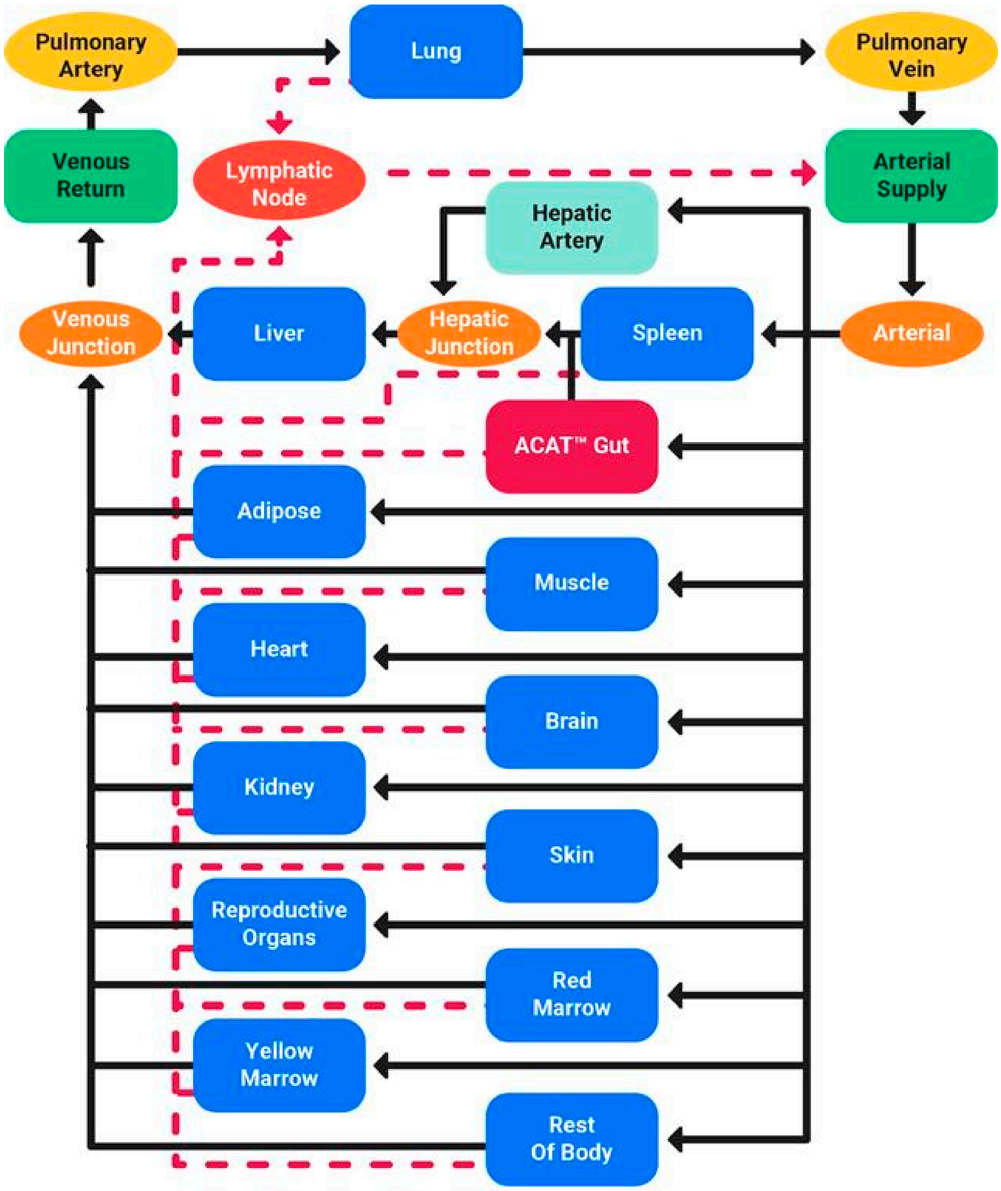

Figure S1: Schematic representation of physiologically based pharmacokinetic model of disposition of biologic drugs. All major organs are connected in an anatomical fashion with plasma flow represented by black solid arrows and lymph flow by red dashed arrows. Lymph node collects lymphatic drainage from organs and lymph fluid is returned to systemic circulation.

Each organ in the PBPK model is divided into three major compartments, representing the vascular, endosomal, and interstitial spaces. The endosomal space of each organ is further subdivided into three sub-compartments as shown in Figure S2.

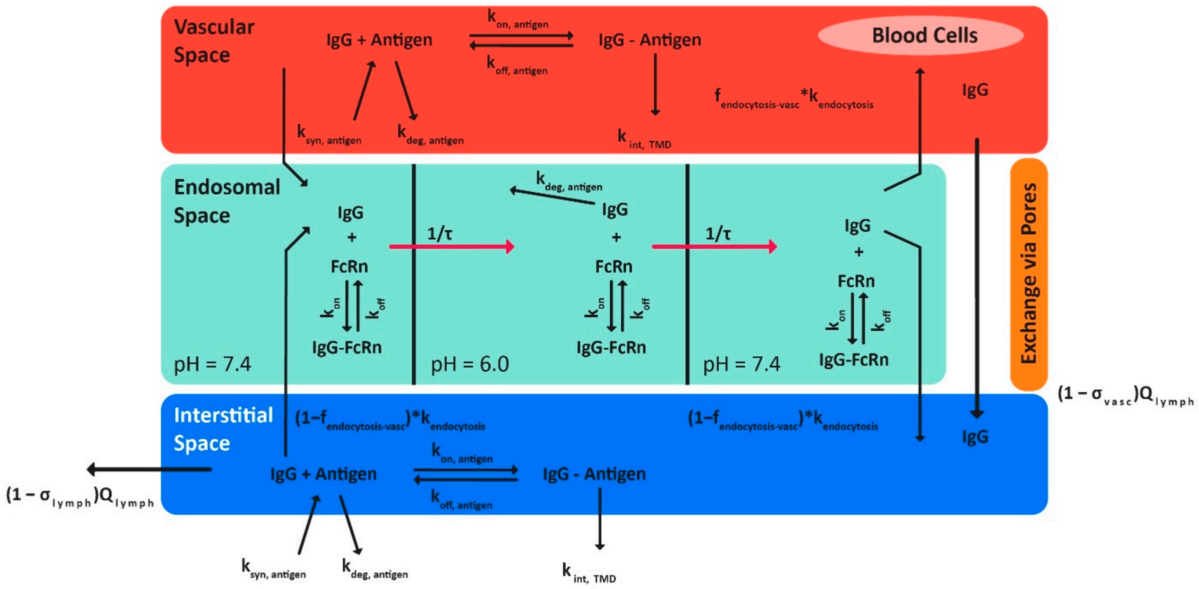

Figure S2: Schematic representation of individual tissue compartments. Model used in this research project also includes target-mediated disposition model in vascular space.

Convective transport and fluid-phase endocytosis are the two main mechanisms related to the uptake of mAbs into the tissue.

Convective transport: Convective transport through the paracellular pores in the vascular endothelium and lymphatic endothelium accounts for passive transfer of mAb from plasma into the tissue interstitial space and from the tissue interstitial space into the lymphatic node, respectively. The convective transport rate is calculated using the vascular reflection coefficient (σ_V) and lymph reflection coefficient (σ_L) for mAb as shown in equation set 1.

$$Vascular - to - Interstitial - Transfer - Rate = (1 - \sigma v) \times Cv \times L$$
$$Interstitial - to - Lymph - Transfer - Rate = (1 - \sigma L) \times Ci \times L \quad \text{Equation S1}$$

where L is the tissue lymph flow, C_V is the mAb concentration in the vascular space, and C_i is the mAb concentration in the interstitial space.

For the vascular reflection coefficient of each individual tissue, the muscle σ_v is used as a reference. σ_v in remaining tissues are calculated by scaling muscle σ_v using tissue specific parameter: relative vascular leakage, $f_{leakage}$. For muscle (as the reference tissue) and number of other tissues with similar integrity of blood capillaries, $f_{leakage} = 1$. For tissues with tighter endothelial walls, $f_{leakage} > 1$ (default value is 1.5 for brain). $F_{leakage} < 1$ (default value is 0.7) for tissues with more leaky endothelial walls. For a given tissue, the final σ_v is calculated as shown in Equation S2.

$$\sigma v = \sigma v, muscle \times fleakage;\ fleakage < 1$$
$$\sigma v = \sigma v, muscle + (1 - \sigma v, muscle) \times (fleakage - 1);\ fleakage \geq 1 \qquad \text{Equation S2}$$

Fluid phase endocytosis: mAbs can be taken up into the endosomal compartment from the vascular and interstitial spaces via fluid phase endocytosis. The rate of mAb uptake into endosomes is calculated as:

$$mAb - Endocytosis - Rate = R1 \times FR \times Cv \times Vendo + R1 \times (1 - FR) \times Ci \times Vendo \qquad \text{Equation S3}$$

where R1 is total endocytosis uptake rate by endothelial cells, V_{endo} is the volume of the endosomal space, and *FR* is the fraction of the total endocytosis uptake rate coming from the vascular space (remainder of the endocytosis uptake rate is from the interstitial space).

mAb-FcRn binding: After uptake into the endosomal space, mAb will bind to the neonatal Fc receptor (FcRn) in acidified endosomal space. At acidic pH, mAb (e.g. IgG) binds to FcRn with high affinity. As the endosomes fuse back with the cell membrane, the mAb-FcRn complex is recycled back to the vascular or interstitial cell surface where the mAb dissociates from the FcRn due to much lower binding affinity at pH 7.4. Meanwhile, the unbound mAb is delivered from endosomes to lysosomal compartments and undergoes degradation [1]. The binding of mAb to FcRn is modeled by the pH-dependent association rate constant (K_{on}) and dissociation rate constant (K_{off}).
To account for changing endosomal pH (and resulting differences in mAb binding to FcRn) throughout the endocytosis/exocytosis process, the endosome is split into three sub-compartments. The transit time of each endosomal sub-compartment is represented by τ. The first endosomal sub-compartment, which represents the early endosomes (just after endocytosis), has pH = 7.4. Mass balance equations for mAb and free FcRn in the first endosomal sub-compartment are described by Equations S4 through S6:

Free mAb:

$$V_{endo-1}\frac{dC_{endo-1f}}{dt} = R1 \times FR \times C_v \times V_{endo} + R1 \times (1 - FR) \times C_i \times V_{endo} - K_{on(7.4)} \times FcRn_{endo-1f} \times C_{endo-1f} \times V_{endo-1} + K_{off(7.4)} \times C_{endo-1b} \times V_{endo-1} - \frac{1}{\tau} \times C_{endo-1f} \times V_{endo-1}$$

Equation S4

Bound mAb:

$$V_{endo-1}\frac{dC_{endo-1b}}{dt} = R2 \times C_{endo-3B} \times V_{endo} + K_{on(7.4)} \times FcRn_{endo-1f} \times C_{endo-1f} \times V_{endo-1} - K_{off(7.4)} \times C_{endo-1b} \times V_{endo-1} - \frac{1}{\tau} \times C_{endo-1b} \times V_{endo-1}$$

Equation S5

Free FcRn:

$$V_{endo-1}\frac{dFcRn_{endo-1f}}{dt} = R2 \times FcRn_{endo-3f} \times V_{endo} - K_{on(7.4)} \times FcRn_{endo-1f} \times C_{endo-1f} \times V_{endo-1} + K_{off(7.4)} \times C_{endo-1B} \times V_{endo-1} - \frac{1}{\tau} \times FcRn_{endo-1f} \times V_{endo-1}$$

Equation S6

The activity of vascular ATPase (V-ATPase) will acidify the endosomal space. The second endosomal sub-compartment represents this acidic stage with pH = 6.0. Certain mAb (e.g. IgG) may have higher binding affinity to FcRn at this pH. The unbound mAb at this stage is delivered to lysosomes and is degraded as described by the rate constant K_{deg}. Mass balance equations for mAb and free FcRn in the second endosomal sub-compartment are described by Equations S7 through S9:

Free mAb:

$$V_{endo-2}\frac{dC_{endo-2f}}{dt} = \frac{1}{\tau} \times C_{endo-1f} \times V_{endo-1} - K_{on(6.0)} \times FcRn_{endo-2f} \times C_{endo-2f} \times V_{endo-2} + K_{off(6.0)} \times C_{endo-2b} \times V_{endo-2} - \frac{1}{\tau} \times C_{endo-2f} \times V_{endo-2} - K_{deg} \times C_{endo-2f} \times V_{endo-2}$$

Equation S7

Bound mAb:

$$V_{endo-2}\frac{dC_{endo-2b}}{dt} = \frac{1}{\tau} \times C_{endo-1b} \times V_{endo-1} + K_{on(6.0)} \times FcRn_{endo-2f} \times C_{endo-2f} \times V_{endo-2} - K_{off(6.0)} \times C_{endo-2b} \times V_{endo-2} - \frac{1}{\tau} \times C_{endo-2b} \times V_{endo-2}$$

Equation S8

Free FcRn:

$$V_{endo-2}\frac{dFcRn_{endo-2f}}{dt} = \frac{1}{\tau} \times FcRn_{endo-1f} \times V_{endo-1} - K_{on(7.4)} \times FcRn_{endo-2f} \times C_{endo-2f} \times V_{endo-2} + K_{off(7.4)} \times C_{endo-2B} \times V_{endo-2} - \frac{1}{\tau} \times FcRn_{endo-2f} \times V_{endo-2}$$

Equation S9

The late endosomes fuse back to the cell membrane where the pH changes to 7.4. Due to lower binding affinity at this pH, mAb-FcRn complexes dissociate and the free mAb is recycled back to vascular and interstitial spaces. The recycling rate is defined as R2. Mass balance equations for mAb and free FcRn in the third endosomal sub-compartment are described by Equations S10 through S12:

Free mAb:

$$V_{endo-3}\frac{dC_{endo-3f}}{dt} = \frac{1}{\tau} \times C_{endo-2f} \times V_{endo-2} - K_{on(7.4)} \times FcRn_{endo-3f} \times C_{endo-3f} \times V_{endo-3} + K_{off(7.4)} \times C_{endo-3b} \times V_{endo-3} - R2 \times C_{endo-3f} \times V_{endo}$$

Equation S10

Bound mAb:

$$V_{endo-3}\frac{dC_{endo-3b}}{dt} = \frac{1}{\tau} \times C_{endo-2b} \times V_{endo-2} + K_{on(7.4)} \times FcRn_{endo-3f} \times C_{endo-3f} \times V_{endo-3} - K_{off(7.4)} \times C_{endo-3b} \times V_{endo-3} - R2 \times C_{endo-3B} \times V_{endo}$$

Equation S11

Free FcRn:

$$V_{endo-3}\frac{dFcRn_{endo-3f}}{dt} = \frac{1}{\tau} \times FcRn_{endo-2f} \times V_{endo-2} - K_{on(7.4)} \times FcRn_{endo-3f} \times C_{endo-3f} \times V_{endo-3} + K_{off(7.4)} \times C_{endo-3B} \times V_{endo-3} - R2 \times FcRn_{endo-3f} \times V_{endo}$$

Equation S12

In Equations S4 through S12, $C_{endo-1f}$, $C_{endo-2f}$ and $C_{endo-3f}$ represent unbound (free) mAb concentrations in individual endosomal sub-compartments; $C_{endo-1b}$, $C_{endo-2b}$ and $C_{endo-3b}$ are concentrations of FcRn-bound mAb in each sub-compartment; K_{deg} is the endosomal degradation rate constant; $FcRn_{endo-1f}$, $FcRn_{endo-2f}$ and $FcRn_{endo-3f}$ are free FcRn concentrations in individual endosomal sub-compartments; and $K_{on(7.4)}$, $K_{on(6.0)}$, $K_{off(7.4)}$, and $K_{off(6.0)}$ are the association and dissociation rate constants for mAb binding to FcRn at pH = 7.4 and 6.0.

Endogenous IgG: Within the endosomal space, the competition for binding to FcRn between endogenous IgG and the therapeutic mAb can also be included in the model. In each individual organ, the mechanisms related to the endogenous IgG transport between its sub-compartments (vascular, endosomal, and interstitial spaces) are similar to those of exogenous mAb as presented in Figure S2.

Target Mediated Distribution and Elimination: The biologic module used in this research program incorporates a target mediated disposition model for surface and circulating target to include the influence of specific antigen–mAb interactions on mAb disposition.

At steady state, the antigen concentration is described by Equation S13:

$$Ksyn, antigen = Kdeg, antigen \times Cantigen, t = 0$$ Equation S13

In the interstitial and/or vascular spaces, mAb can bind to the specific antigen. The antigen–mAb association rate, dissociation rate and the internalization rate of the antigen–mAb complex are described by Equation S14.

$$Association - Rate \ = \ Kon, antigen \times (Cantigen - Cib) \times Cif \times Vi$$
$$Dissociation - Rate \ = \ Koff, antigen \times Cib \times Vi$$ Equation S14
$$Complex - Elimination - Rate \ = \ Kint, TMD \times Cib \times Vi$$

The change in antigen concentration due to mAb binding and antigen–mAb complex elimination is described by Equation S15:

$$V_{idCantigendt} = K_{syn,\, antigen} \times V_i - K_{deg,\, antigen} \times (C_{antigen} - C_{ib}) \times V_i - K_{int,\, TMD} \times C_{ib} \times V_i$$
Equation S15

In Equations S13 through S15, $K_{syn,antigen}$ is the synthesis rate of antigen; $K_{deg,antigen}$ is the degradation rate of antigen; $K_{int,TMD}$ is the internalization rate of antigen-mAb complex; $K_{on,antigen}$ and $K_{off,antigen}$ are the association and dissociation rate constants, respectively, between mAb and the antigen; $C_{antigen}$ is the antigen concentration in the interstitial space; V_i is the volume of interstitial space; and C_{if} and C_{ib} are the free and antigen-bound mAb concentrations in the interstitial space.

Supplementary Material S2

Table S1: List of studies used for development and qualification of PBPK models in current study.

Drug	Target	Age (years)	N	BW (kgs)	Disease state	Dose (mg/kg)	Gender	References	Development / validation	Study Code
**Bevacizumab**	VEGF-A	27.6	119	77.49	Healthy	1	Male	[2]	Development	Bev.Ad.H.1
		27	91	80	Healthy	1	Male	[3]	Validation	Bev.Ad.H.2
		31	111	80	Healthy	1	Male	[4]	Validation	Bev.Ad.H.3
		23	43	76.5	Healthy	1	Male	[5]	Validation	Bev.Ad.H.4
		39.5	40	78.97	Healthy	3	Male	[6]	Validation	Bev.Ad.H.5
		39.8	114	79.28	Healthy	3	Male	[7]	Validation	Bev.Ad.H.6
		56	61	89.01 (G+)	Cancer	5	Male	[8]	Validation	Bev.Ad.P.1
		51	25	79.51 (G+)	Cancer	0.1 to 10	Female	[9]	Development	Bev.Ad.P.2
		13	8	53.65 (G+)	Cancer	5, 15	Male	[10,11]	Validation	Bev.Ped.P
**Infliximab**	TNF-alpha	40	159	80.5	Healthy	5	Male	[12]	Development	Inf.Ad.H.1
		41	71	78.1	Healthy	5	Male	[13]	Validation	Inf.Ad.H.2
		30 (G+)	NA	70 (G+)	RA	5, 10, 20	Male	[14,15]	Development	Inf.Ad.P.1
		47	14	79.57 (G+)	RA	3	Female	[16,17]	Validation	Inf.Ad.P.2
		56.3	15	78.86 (G+)	RA	10	Female	[16,17]	Validation	Inf.Ad.P.3
		51.5	101	73.1	RA	3	Female	[18]	Validation	Inf.Ad.P.4
		51	11	87	PS	5	Male	[19,20]	Validation	Inf.Ad.P.5
		35	11	96	PS	10	Male	[19,20]	Validation	Inf.Ad.P.6
		41.2	482	78.8	UC	5, 10	Male	[21]	Validation	Inf.Ad.P.7
		37.5	580	71.1	CD	5	Female	[22]	Validation	Inf.Ad.P.8
		13	112	43.8	CD	5	Male	[22]	Validation	Inf.Ped.P.1
		4 to 19	141	55.8	IBD	5 to 18	Male	[23–25]	Validation	Inf.Ped.P.2
**Atezolizumab**	PD-L1	61	277	88.43 (G+)	Cancer	1 to 20	Male	[26]	Development	Ate.Ad.P
		2 to 16	72	8.7 to 154	Cancer	15 (max 1200 mg)	Male	[27,28]	Validation	Ate.Ped.P

N: Number of individuals
NA: Not available
RA: Rheumatoid arthritis
PS: Psoriasis
AS: Ankylosing spondylitis
UC: Ulcerative colitis
CD: Crohn's disease
IBD: Inflammatory bowel disease

G+: GastroPlus default value

Supplementary Material S3

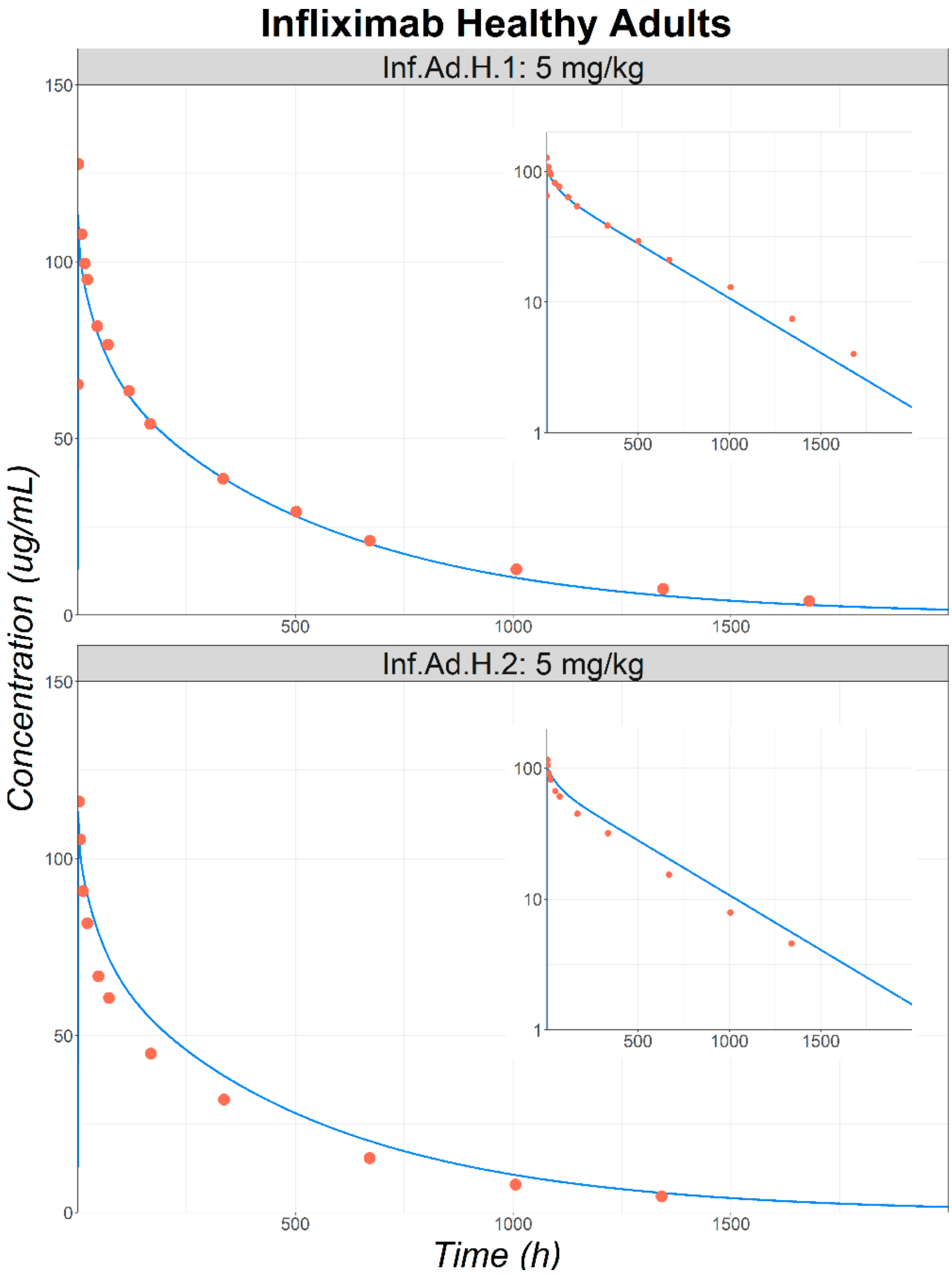

Figure S3: Observed (circle) and simulated (lines) infliximab plasma concentration–time course in healthy subjects following IV administration (5mg/kg). Study Inf.Ad.H.1 was used for model development and study Inf.Ad.H.2 was used for external validation.

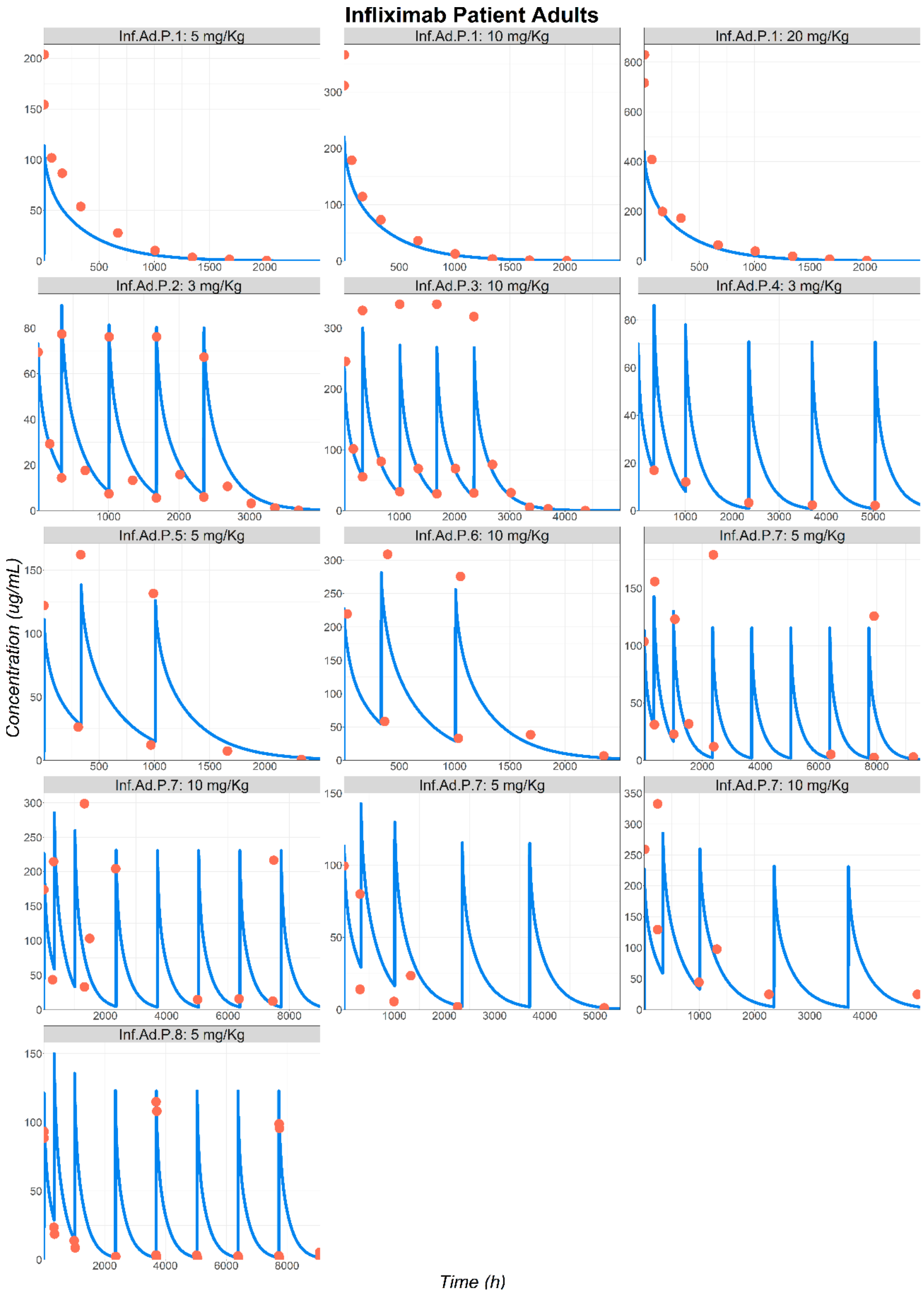

Figure S4: Observed (circle) and simulated (lines) infliximab plasma concentration–time course in patients following IV administration. Study Inf.Ad.P.1 was used for model development and studies Inf.Ad.P.1 to Inf.Ad.P.8 were used for external validation.

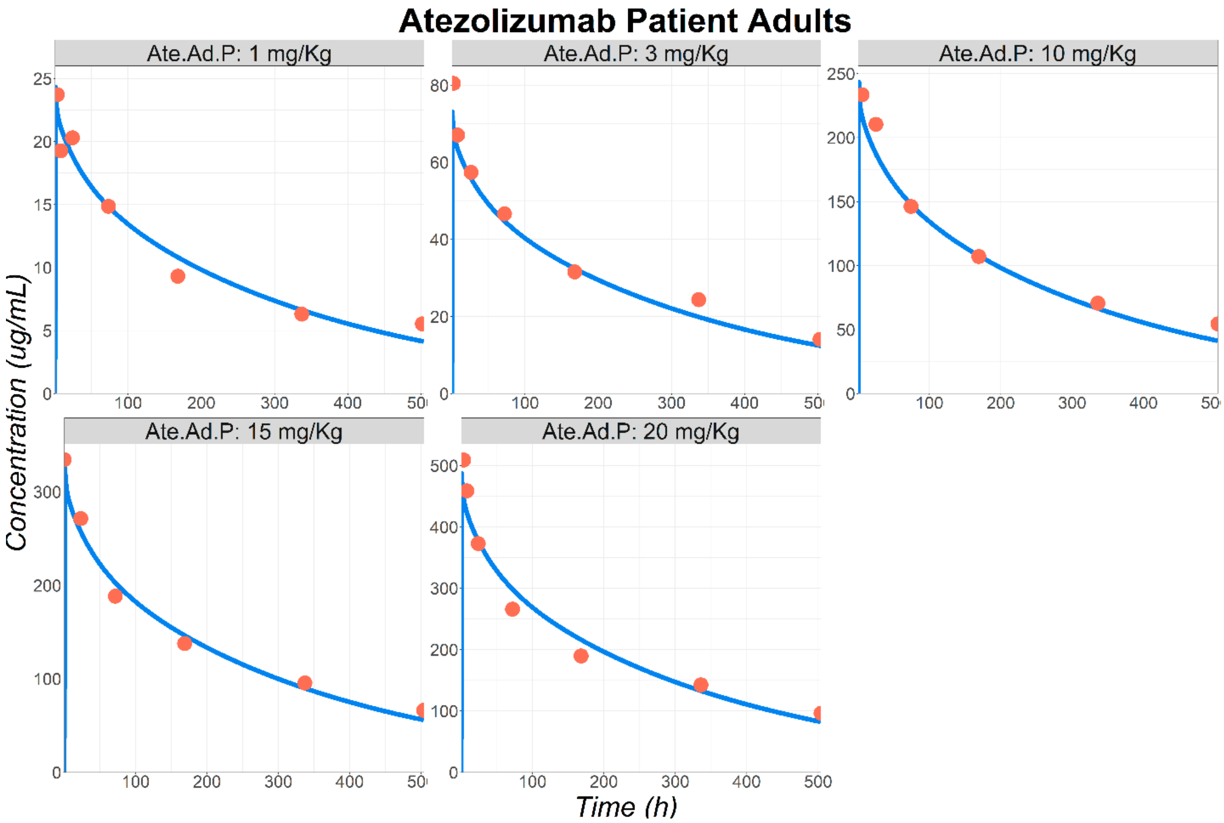

Figure S5: Observed [26] (circles) and simulated (lines) atezolizumab plasma concentration–time course in patients following IV administration. Strength of 15 mg/kg was used for model development and other strengths were used for external validation.

Table S2: Cmax and AUC0-t, observed, simulated, and prediction fold error for studies used in baseline PBPK model validation.

**Study Code**	**dose (mg)**	***Cmax (ug/mL)***			***AUC_{0-t} (ug·h/mL)***		
		Observed	***Simulated***	***Fold Error***	***Observed***	***Simulated***	***Fold Error***
Bev.Ad.H.1	1	23	22.9	**1.00**	7355	7740	**1.05**
Bev.Ad.H.2	1	24.5	22	**0.90**	6603	6811	**1.03**
Bev.Ad.H.3	1	25	22	**0.88**	8076.2	7329.3	**0.91**
Bev.Ad.H.4	1	25	22.7	**0.91**	6954.2	6748.4	**0.97**
Bev.Ad.H.5	3	83.5	66.3	**0.79**	33780	23230	**0.69**
Bev.Ad.H.6	3	41.1	66.3	**1.61**	19960	23110	**1.16**
Inf.Ad.H.1	5	127.6	108.4	**0.85**	41360	36260	**0.88**
Inf.Ad.H.2	5	116.1	113.2	**0.97**	30850	38280	**1.24**
Ate.Ad.P	1	23.7	24.3	**1.03**	4761.1	4823.8	**1.01**
Ate.Ad.P	3	80.4	73.0	**0.91**	15700	14470	**0.92**
Ate.Ad.P	10	233.4	243.3	**1.04**	51090	48220	**0.94**
Ate.Ad.P	15	334.3	330.2	**0.99**	67050	65430	**0.98**
Ate.Ad.P	20	508.9	486.7	**0.96**	94880	96410	**1.02**

Supplementary Material S4

Table S3: Parameter sensitivity analysis settings to assess effect of FcRn on mAbs PK. PSA was done using Study Inf.Ped.P.1 (baseline simulation results presented in Figure 5 of main publication)

Parameter Name	***Baseline***	***Lower Limit***	***Upper Limit***
FcRnDensity-Liver (microM)	13	6.5	26
FcRnDensity-RestOfBody (microM)	1.66	0.83	3.32
FcRnDensity-YellowMarrow (microM)	1.66	0.83	3.32
FcRnDensity-RedMarrow (microM)	1.66	0.83	3.32
FcRnDensity-ReproOrg (microM)	1.66	0.83	3.32
FcRnDensity-Skin (microM)	7.3	3.65	14.6
FcRnDensity-Kidney (microM)	7.76	3.88	15.52
FcRnDensity-Brain (microM)	1.66	0.83	3.32
FcRnDensity-Heart (microM)	4.36	2.18	8.72
FcRnDensity-Spleen (microM)	3.81	1.905	7.62
FcRnDensity-Muscle (microM)	1.66	0.83	3.32
FcRnDensity-Adipose (microM)	1.66	0.83	3.32
FcRnDensity-Lung (microM)	5.83	2.915	11.66
FcRn Koff pH 6	500	250	1000
FcRn Kon pH 6	8000	4000	16000

Table S4: Parameter sensitivity analysis results for Cmax (Study Inf.Ped.P.1)

	Cmax (ug/mL)									
**Scaling Factor =**	*0.5*	*0.58*	*0.68*	*0.79*	*0.92*	*1.08*	*1.26*	*1.47*	*1.71*	*2*
FcRnDensity-Liver (microM)	142.9	142.9	143	143	143	143	143	143	143	143
FcRnDensity-RestOfBody (microM)	141.8	142.1	142.4	142.7	142.9	143.1	143.3	143.4	143.5	143.7
FcRnDensity-YellowMarrow (microM)	142.6	142.7	142.8	142.9	143	143	143.1	143.2	143.2	143.2
FcRnDensity-RedMarrow (microM)	142.7	142.8	142.9	142.9	143	143	143.1	143.1	143.1	143.1
FcRnDensity-ReproOrg (microM)	143	143	143	143	143	143	143	143	143	143
FcRnDensity-Skin (microM)	142.9	142.9	142.9	143	143	143	143	143	143	143.1
FcRnDensity-Kidney (microM)	143	143	143	143	143	143	143	143	143	143
FcRnDensity-Brain (microM)	142.6	142.7	142.8	142.9	143	143	143.1	143.1	143.2	143.2
FcRnDensity-Heart (microM)	143	143	143	143	143	143	143	143	143	143
FcRnDensity-Spleen (microM)	143	143	143	143	143	143	143	143	143	143
FcRnDensity-Muscle (microM)	138.6	139.7	140.8	141.7	142.6	143.4	144.1	144.7	145.2	145.7
FcRnDensity-Adipose (microM)	140.4	141.1	141.7	142.3	142.8	143.2	143.6	143.9	144.2	144.5
FcRnDensity-Lung (microM)	142.9	143	143	143	143	143	143	143	143	143
FcRn Koff pH 6	153.4	151.3	149.1	146.8	144.3	141.7	139.1	136.4	133.7	131.2
FcRn Kon pH 6	128.5	131.4	134.5	137.8	141.2	144.8	148.4	151.9	155.4	158.7

Table S5: Parameter sensitivity analysis results for AUC (Study Inf.Ped.P.1)

	AUC (g-h/mL)									
**Scaling Factor =**	*0.5*	*0.58*	*0.68*	*0.79*	*0.92*	*1.08*	*1.26*	*1.47*	*1.71*	*2*
FcRnDensity-Liver (microM)	236	236	236	236	236	236	236	237	237	237
FcRnDensity-RestOfBody (microM)	227	229	232	234	235	237	238	240	241	242
FcRnDensity-YellowMarrow (microM)	233	234	235	235	236	237	237	238	238	238
FcRnDensity-RedMarrow (microM)	234	235	235	236	236	236	237	237	237	237
FcRnDensity-ReproOrg (microM)	236	236	236	236	236	236	236	236	236	236
FcRnDensity-Skin (microM)	235	236	236	236	236	236	236	237	237	237
FcRnDensity-Kidney (microM)	236	236	236	236	236	236	236	236	236	236
FcRnDensity-Brain (microM)	233	234	235	235	236	237	237	237	238	238
FcRnDensity-Heart (microM)	236	236	236	236	236	236	236	236	236	236
FcRnDensity-Spleen (microM)	236	236	236	236	236	236	236	236	236	236
FcRnDensity-Muscle (microM)	203	211	219	226	233	239	245	250	255	260
FcRnDensity-Adipose (microM)	216	221	226	230	234	238	241	244	247	249
FcRnDensity-Lung (microM)	236	236	236	236	236	236	236	236	236	237
FcRn Koff pH 6	339	315	291	269	247	226	207	189	173	158
FcRn Kon pH 6	143	159	177	198	223	251	284	322	365	415

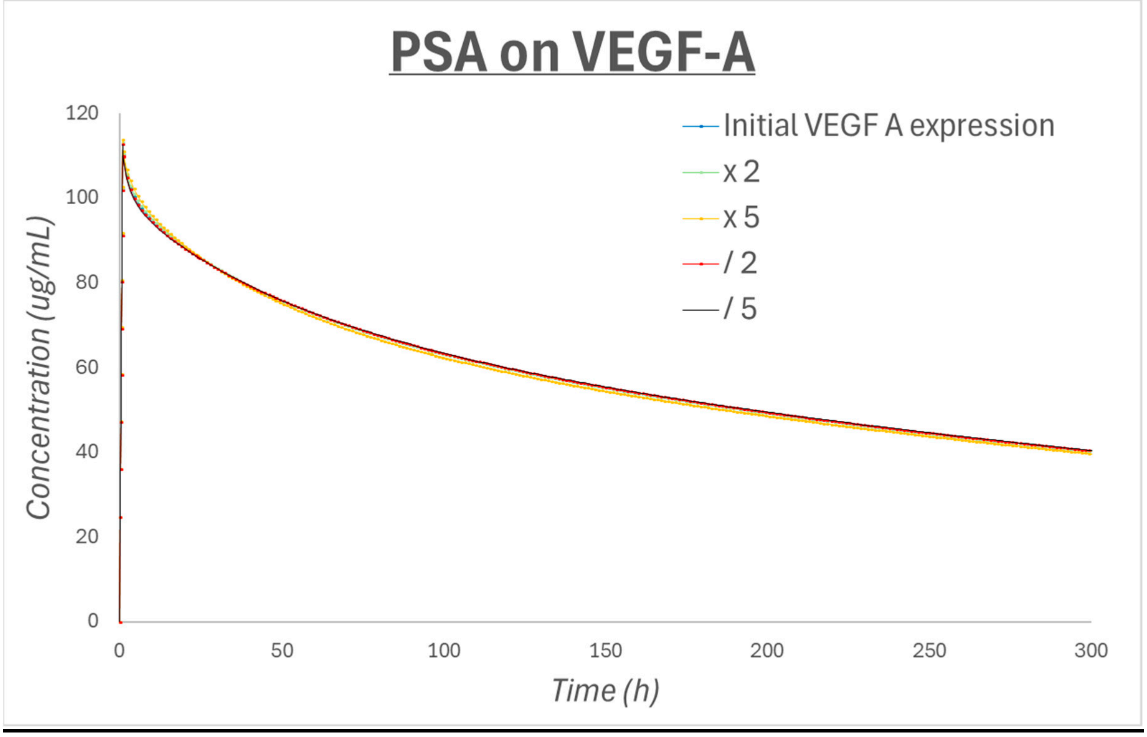

Figure S6: Simulated BEV plasma concentration–time course in pediatric patients following IV administration of 5 mg/kg (Study Bev.Ped.P) with varying concentration of circulating VEGF-A.

References

1. Ober RJ, Martinez C, Vaccaro C, Zhou J, Ward ES. Visualizing the Site and Dynamics of IgG Salvage by the MHC Class I-Related Receptor, FcRn. The Journal of Immunology. 2004 Feb 15;172(4):2021–9.

2. Demarchi M, Coliat P, Barthelemy P, Schott R, BenAbdelghani M, Kim M, et al. A randomized phase I study comparing the pharmacokinetics of a bevacizumab (HD204) biosimilar to European Union- and United States of America-sourced bevacizumab. Handelsman DJ, editor. PLoS ONE. 2021 Sep 23;16(9):e0248222.

3. Hettema W, Wynne C, Lang B, Altendorfer M, Czeloth N, Lohmann R, et al. A randomized, single-blind, Phase I trial (INVICTAN-1) assessing the bioequivalence and safety of BI 695502, a bevacizumab biosimilar candidate, in healthy subjects. Expert Opinion on Investigational Drugs. 2017 Aug 3;26(8):889–96.

4. Hummel M, Bosje T, Shaw A, Liu MS, Barve A, Kothekar M, et al. A pharmacokinetics study of proposed bevacizumab biosimilar MYL-1402O vs EU-bevacizumab and US-bevacizumab. J Cancer Res Clin Oncol. 2022 Feb;148(2):487–96.

5. Wu X, Wynne C, Xu C, Gan Y, Wang C, Thomas BE, et al. A Global Phase I Clinical Study Comparing the Safety and Pharmacokinetics of Proposed Biosimilar BAT1706 and Bevacizumab (Avastin®) in Healthy Male Subjects. BioDrugs. 2019 Jun;33(3):335–42.

6. Shin D, Lee YJ, Choi J, Lee D, Park M, Petkova M. A phase I, randomized, single-dose pharmacokinetic study comparing sb8 (bevacizumab biosimilar) with reference bevacizumab in healthy volunteers. Cancer Chemother Pharmacol. 2020 Oct;86(4):567–75.

7. Sinn A, García-Alvarado F, Gonzalez V, Huerga C, Bullo F. A randomized, double blind, single dose, comparative study of the pharmacokinetics, safety and immunogenicity of MB02 (bevacizumab biosimilar) and reference bevacizumab in healthy male volunteers. Brit J Clinical Pharma. 2022 Mar;88(3):1063–73.

8. Romera A. Bevacizumab biosimilar BEVZ92 versus reference bevacizumab in combination with FOLFOX or FOLFIRI as first-line treatment for metastatic colorectal cancer: a multicentre, open-label, randomised controlled trial. :11.

9. Gordon MS, Margolin K, Talpaz M, Sledge GW, Holmgren E, Benjamin R, et al. Phase I safety and pharmacokinetic study of recombinant human anti-vascular endothelial growth factor in patients with advanced cancer. J Clin Oncol. 2001 Feb 1;19(3):843–50.

10. Basu S, Lien YT (Kayla), Vozmediano V, Schlender JF, Eissing T, Schmidt S, et al. Physiologically Based Pharmacokinetic Modeling of Monoclonal Antibodies in Pediatric Populations Using PK-Sim. Front Pharmacol. 2020 Jun 11;11:868.

11. Bender JLG, Adamson PC, Reid JM, Xu L, Baruchel S, Shaked Y, et al. Phase I Trial and Pharmacokinetic Study of Bevacizumab in Pediatric Patients With Refractory Solid Tumors: A Children's Oncology Group Study. JCO. 2008 Jan 20;26(3):399–405.

12. Shin D, Kim Y, Kim YS, Körnicke T, Fuhr R. A Randomized, Phase I Pharmacokinetic Study Comparing SB2 and Infliximab Reference Product (Remicade(®)) in Healthy Subjects. BioDrugs. 2015 Dec;29(6):381–8.

13. Park W, Lee SJ, Yun J, Yoo DH. Comparison of the pharmacokinetics and safety of three formulations of infliximab (CT-P13, EU-approved reference infliximab and the US-licensed reference infliximab) in healthy subjects: a randomized, double-blind, three-arm, parallel-group, single-dose, Phase I study. Expert Review of Clinical Immunology. 2015 Sep 28;11(sup1):25–31.

14. Yuan D, Rode F, Cao Y. A Minimal Physiologically Based Pharmacokinetic Model with a Nested Endosome Compartment for Novel Engineered Antibodies. AAPS J. 2018 Mar 14;20(3):48.

15. Kavanaugh A, St Clair EW, McCune WJ, Braakman T, Lipsky P. Chimeric anti-tumor necrosis factor-alpha monoclonal antibody treatment of patients with rheumatoid arthritis receiving methotrexate therapy. J Rheumatol. 2000 Apr;27(4):841–50.

16. Maini RN, Breedveld FC, Kalden JR, Smolen JS, Davis D, Macfarlane JD, et al. Therapeutic efficacy of multiple intravenous infusions of anti-tumor necrosis factor alpha monoclonal antibody combined with low-dose weekly methotrexate in rheumatoid arthritis. Arthritis Rheum. 1998 Sep;41(9):1552–63.

17. Elliott MJ, Maini RN, Feldmann M, Long-Fox A, Charles P, Bijl JA, et al. Repeated therapy with monoclonal antibody to tumour necrosis factor α (cA2) in patients with rheumatoid arthritis. The Lancet. 1994 Oct;344(8930):1125–7.

18. Smolen JS, Choe JY, Prodanovic N, Niebrzydowski J, Staykov I, Dokoupilova E, et al. Safety, immunogenicity and efficacy after switching from reference infliximab to biosimilar SB2 compared with continuing reference infliximab and SB2 in patients with rheumatoid arthritis: results of a randomised, double-blind, phase III transition study. Ann Rheum Dis. 2018 Feb;77(2):234–40.

19. Gottlieb AB, Masuda S, Ramamurthi R, Abdulghani A, Romano P, Chaudhari U, et al. Pharmacodynamic and pharmacokinetic response to anti-tumor necrosis factor-α monoclonal antibody (infliximab) treatment of moderate to severe psoriasis vulgaris. Journal of the American Academy of Dermatology. 2003 Jan;48(1):68–75.

20. Chaudhari U, Romano P, Mulcahy L, Dooley L, Baker D, Gottlieb A. Efficacy and safety of infliximab monotherapy for plaque-type psoriasis: a randomised trial. The Lancet. 2001 Jun;357(9271):1842–7.

21. Fasanmade AA, Adedokun OJ, Ford J, Hernandez D, Johanns J, Hu C, et al. Population pharmacokinetic analysis of infliximab in patients with ulcerative colitis. Eur J Clin Pharmacol. 2009;65(12):1211–28.

22. Fasanmade AA, Adedokun OJ, Blank M, Zhou H, Davis HM. Pharmacokinetic Properties of Infliximab in Children and Adults with Crohn's Disease: A Retrospective Analysis of Data from 2 Phase III Clinical Trials. Clinical Therapeutics. 2011 Jul;33(7):946–64.

23. Chang HP, Shakhnovich V, Frymoyer A, Funk RS, Becker ML, Park KT, et al. A population physiologically-based pharmacokinetic model to characterize antibody disposition in pediatrics and evaluation of the model using infliximab. British Journal of Clinical Pharmacology [Internet]. 2021 [cited 2022 Jan 14];n/a(n/a). Available from: http://onlinelibrary.wiley.com/doi/abs/10.1111/bcp.14963

24. Funk RS, Shakhnovich V, Cho YK, Polireddy K, Jausurawong T, Gress K, et al. Factors associated with reduced infliximab exposure in the treatment of pediatric autoimmune disorders: a cross-sectional prospective convenience sampling study. Pediatr Rheumatol. 2021 Dec;19(1):62.

25. Piester T, Frymoyer A, Christofferson M, Yu H, Bass D, Park KT. A Mobile Infliximab Dosing Calculator for Therapy Optimization in Inflammatory Bowel Disease. Inflammatory Bowel Diseases. 2018 Jan 18;24(2):227–34.

26. Herbst RS, Soria JC, Kowanetz M, Fine GD, Hamid O, Gordon MS, et al. Predictive correlates of response to the anti-PD-L1 antibody MPDL3280A in cancer patients. Nature. 2014 Nov 27;515(7528):563–7.

27. Huang W, Stader F, Chan P, Shemesh CS, Chen Y, Gill KL, et al. Development of a Pediatric Physiologically-Based Pharmacokinetic Model to Support Recommended Dosing of Atezolizumab in Children with Solid Tumors. Front Pharmacol. 2022 Sep 26;13:974423.

28. Shemesh CS, Chanu P, Jamsen K, Wada R, Rossato G, Donaldson F, et al. Population pharmacokinetics, exposure-safety, and immunogenicity of atezolizumab in pediatric and young adult patients with cancer. j immunotherapy cancer. 2019 Dec;7(1):314.
